# Automated Enrichment, Transduction, and Expansion of Clinical-Scale CD62L^+^ T Cells for Manufacturing of Gene Therapy Medicinal Products

**DOI:** 10.1089/hum.2016.091

**Published:** 2016-10-01

**Authors:** Christoph Priesner, Krasimira Aleksandrova, Ruth Esser, Nadine Mockel-Tenbrinck, Jana Leise, Katharina Drechsel, Michael Marburger, Andrea Quaiser, Lilia Goudeva, Lubomir Arseniev, Andrew D. Kaiser, Wolfgang Glienke, Ulrike Koehl

**Affiliations:** ^1^ Cellular Therapy Center, Institute of Cellular Therapeutics, Hannover Medical School, Hannover, Germany; ^2^ GMP Development Unit, Institute of Cellular Therapeutics, Integrated Research and Treatment Center for Transplantation, Hannover Medical School, Hannover, Germany; ^3^ Miltenyi Biotec, Bergisch Gladbach, Germany.; ^4^ Institute of Transfusion Medicine, Hannover Medical School, Hannover, Germany

## Abstract

Multiple clinical studies have demonstrated that adaptive immunotherapy using redirected T cells against advanced cancer has led to promising results with improved patient survival. The continuously increasing interest in those advanced gene therapy medicinal products (GTMPs) leads to a manufacturing challenge regarding automation, process robustness, and cell storage. Therefore, this study addresses the proof of principle in clinical-scale selection, stimulation, transduction, and expansion of T cells using the automated closed CliniMACS^®^ Prodigy system. Naïve and central memory T cells from apheresis products were first immunomagnetically enriched using anti-CD62L magnetic beads and further processed freshly (*n* = 3) or split for cryopreservation and processed after thawing (*n* = 1). Starting with 0.5 × 10^8^ purified CD3^+^ T cells, three mock runs and one run including transduction with green fluorescent protein (GFP)-containing vector resulted in a median final cell product of 16 × 10^8^ T cells (32-fold expansion) up to harvesting after 2 weeks. Expression of CD62L was downregulated on T cells after thawing, which led to the decision to purify CD62L^+^CD3^+^ T cells freshly with cryopreservation thereafter. Most important in the split product, a very similar expansion curve was reached comparing the overall freshly CD62L selected cells with those after thawing, which could be demonstrated in the T cell subpopulations as well by showing a nearly identical conversion of the CD4/CD8 ratio. In the GFP run, the transduction efficacy was 83%. In-process control also demonstrated sufficient glucose levels during automated feeding and medium removal. The robustness of the process and the constant quality of the final product in a closed and automated system give rise to improve harmonized manufacturing protocols for engineered T cells in future gene therapy studies.

## Introduction

Significant progress has been made in the last decade by using redirected immune response against advanced cancer, especially for leukemia.^1,2^ Clinical Phase I/II studies have shown the successful treatment with autologous T cells redirected against CD19 leukemic cells by using recombinant chimeric antigen receptors (CARs), which consist of a single-chain variable fragment (scFv) linked to intracellular signaling domains. Long-term remission in those patients with B cell malignancies has been observed.^2,3^ In addition, a broad range of different cancer target antigens under clinical investigation using CAR-expressing T cells redirected against CD20, CD30, CD138, c-Met, EGFRvIII, ErbB2, FAB, GD2, HER2, WT1, PSMA, NY-ESO1, and others have been reviewed.^4–6^ In a similar way, both autologous and allogeneic natural killer (NK) effector cells,^7^ as well as the NK cell line NK-92, can be redirected against cancer. Preclinical experiments have shown this successfully, as reviewed in Glienke *et al*.^8^ and Klingemann *et al*.^9^

To date, clinical-scale expansion and transduction of engineered T cells or other effector cells for Phase I/II trials is a complex multistep hands-on process and a standardized automated manufacturing process observing good manufacturing practice (GMP) conditions is still a challenge. Currently, the use of a closed, automated system—the CliniMACS^®^ Prodigy (Miltenyi Biotec, Bergisch Gladbach, Germany)—has been successfully demonstrated as a short-term process for the purification of CD34^+^ progenitor cells^10,11^ or cytomegalovirus (CMV)-specific T cells,^12^ as well as for long-term NK cell expansion.^13^

In order to prove whether such a system can be used to improve process robustness for more complex manufacturing protocols, this study established and evaluated a GMP-compliant clinical-scale protocol for gene-modified T cells on the Prodigy system, including T cell purification, transduction, and expansion, with final cell harvesting within 2 weeks. As a first step, a CD62L selection from the apheresis product was chosen to generate naïve and central memory T cells for the following transduction and expansion. The present data support both the feasibility of regulatory compliant and harmonized manufacturing protocols for gene therapy trials.

## Material and Methods

### Donor cell preparation and cryopreservation

Peripheral blood apheresis products from healthy donors were obtained from the Institute for Transfusion Medicine of Hannover Medical School (MHH) after donors’ written informed consent. The protocol was approved by the ethics committee (#2830-2015) of Hannover Medical School. For freezing/thawing experiments with respect to CD62L expression, fresh leukapheresis was reformulated in TexMACS^™^ Medium (Miltenyi Biotec) supplemented with 20% heat-inactivated human AB serum (GemCell; Gemini Bioproducts, Bolney, United Kingdom) and 10% (v/v) dimethyl sulfoxide (DMSO; Sigma-Aldrich, Munich, Germany), and stored for 3 days at <–60°C. Samples were thawed and recovered 24, 48, and 72 h before flow cytometric acquisition was performed. After thawing, cells were immediately washed twice in pre-warmed TexMACS^™^ medium without supplements, and plated for recovery with 1 × 10^7^ white blood cells (WBCs)/mL in a 24-well plate.

### Selection, stimulation, transduction, and expansion via the Prodigy system

Enrichment of CD62L^+^ cells, transduction with lentiviral green fluorescent protein (GFP) containing vector, and expansion of cells were performed with the Prodigy system (Miltenyi Biotec), a closed automated GMP cell processing system developed for integrated cell processing from starting material to final cellular product, even for long-term cell cultivation (>12 days).

**Figure 1.**
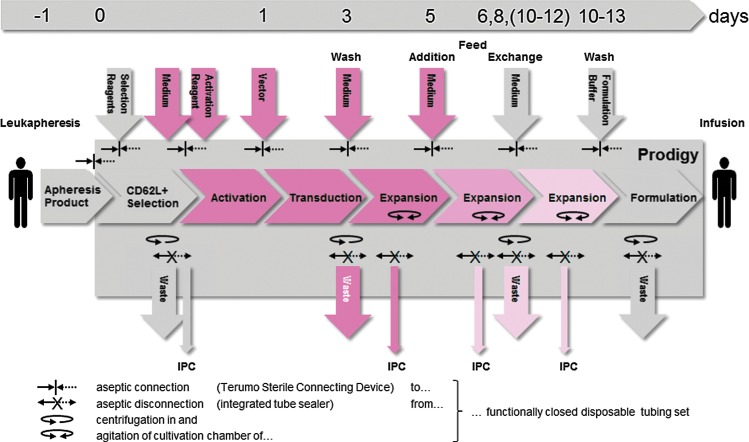
Automated manufacturing of both expanded and genetically modified naïve and central memory T cells with the Prodigy system. The manufacturing process of genetically modified T cells involves the immunomagnetic enrichment of CD62L^+^ cells from unstimulated leukapheresis products followed by polyclonal activation (CD28 and CD3), lentiviral transduction, and subsequent expansion of the target fraction: day 0, immunomagnetic selection and activation; day 1, start of transduction; day 3, removal of TransAct CD3/CD28 Reagent and vector, start of dynamic culture; day 5, feed by addition of medium with AB serum; days 6, 8 (10–12), feed by exchange of increasing volumes of cell-free supernatant by medium without AB serum; between days 10 and 13, harvest and final formulation of cells. The implementation of additional feeding steps depends on the duration of the expansion phase. The fading of the *red* color depicts depletion of AB serum and vector. The integrated process is conducted in a sterile functionally closed disposable tubing system. Sample and waste receptacles as well as inoculum, reagent, and media reservoirs are aseptically dis/connected by heat sealing/welding. IPC, in-process control.

First, CliniMACS^®^ phosphate-buffered saline (PBS)/ethylene-diamine-tetraacetic acid (EDTA) buffer containing 0.5% (w/v) human serum albumin (HSA; 200 g/L; Baxter, Unterschleissheim, Germany) and TexMACS™ GMP medium supplemented with interleukin-7 (IL-7) and IL-15 (both Miltenyi Biotec) and 3% (v/v) of heat-inactivated AB serum (Biochrom, Berlin, Germany) were prepared. According to the instructions on the guided user interface of the Prodigy system, the TS520 tubing set was installed, and reagents, buffer, media, and max. 3 × 10^9^ CD62L^+^ cells (aliquot of the unstimulated apheresis product) were connected to the appropriate ports of the tubing set. The processing was controlled by the Prodigy system's software v1.1.4 and process version v0.8. CliniMACS^®^ CD62L Reagent (Miltenyi Biotec) was used for enrichment, and MACS GMP TransAct CD3 Reagent (1:200) and MACS GMP TransAct CD28 Reagent (1:400) were used for stimulation of 0.5–2 × 10^8^ WBCs according to the manufacturer's instructions (Miltenyi Biotec). Transduction took place on day 1 of cultivation by adding 10 mL of a GFP-containing SIN, VSV-G pseudotyped lentiviral vector with an SFFV promotor (donor 3_GFP_ run) or 10 mL of supplemented TexMACS™ GMP medium in case of mock runs (donor1, donor2_fresh/cryo_). The lentiviral vector was produced using HEK293T cells (ATCC CRL-11268). The supernatant was concentrated and stored at <–60°C until transduction. The vector titer was determined on HT1080 cells (ATCC CCL-121). A multiplicity of infection (MOI) of 1 was used for transduction. After day 5 of cultivation, the concentration of AB serum was reduced using TexMACS™ GMP medium containing IL-7 and IL-15, respectively, but without AB serum for further medium exchange. On day of harvest, cells were formulated in Composol^®^ PS (Fresenius Kabi Deutschland GmbH, Bad Homburg, Germany) containing 2.86% (w/v) HSA (200 g/L, Baxter). A summary of the process is shown in Fig. 1. After CD62L selection, a part of the positive fraction not being used for cell stimulation and expansion was cryopreserved in Composol^®^ PS containing 2.86% (w/v) HSA (as above) with 10% (v/v) DMSO (Cryosure; WAK-Chemie Medical GmbH, Steinbach/Ts., Germany). After cryopreservation in a <–60°C freezer overnight, the cells were stored in the vapor phase above liquid nitrogen at <–140°C. Cryopreserved CD62L selected cells were thawed, washed, and resuspended in TexMACS™ GMP medium with 3% (v/v) heat-inactivated human AB serum (Biochrom), 10 ng/mL MACS^®^ GMP recombinant human IL-7 and 10 ng/mL MACS^®^ GMP recombinant human IL-15 for cultivation in a CO_2_ incubator (37°C, 5% CO_2_) overnight. The T cell transduction (TCT) process was started with stimulation, mock transduction, and expansion (as described above).

### In-process control

A cell count was performed with an automated hematology analyzer (Sysmex KX-21N; Sysmex, Norderstedt, Germany). In addition, the total cell number and viability were analyzed after trypan blue staining in a Neubauer improved counting chamber and independently by flow cytometric analysis as described below.

The glucose concentration of the cell-free culture supernatant was determined with the blood glucose meter Accu Chek Aviva (Roche, Mannheim, Germany). Samples for microbiological testing were taken at different time points during cultivation. Tests for microbiological contamination were performed at the Institute for Medical Microbiology and Hospital Epidemiology of MHH.

Samples for flow cytometric analysis were taken from unmanipulated apheresis, after CD62L selection at the start of cultivation, at different days during culture (days 6, 7, 8, and 9) and at the end of the cell culture (days 10 and 12). Where indicated, samples were diluted with Dulbecco's PBS (Gibco; Life Technologies, Darmstadt, Germany) to a cell concentration <20 × 10^6^/mL. The cells were stained with fluorochrome-conjugated monoclonal antibodies (mAb) according to their manufacturer's instructions. The following mAbs were used: CD4-FITC, CD62L-FITC, and CD20-PE/Cy7 (both BD Bioscience, Heidelberg, Germany); CD62L-PE-Vio770 (Miltenyi Biotec); CD3-APC, CD14-PB, CD45-KO, CD45-PB, CD8-ECD, CD16-, CD56-, and CD45RA-PE, CD45R0-FITC, and all other reagents for flow cytometry (Beckman Coulter, Krefeld, Germany). After 15 min of incubation at ambient temperature in the dark, NH_4_Cl solution was added for erythrocyte lysis or just for consistency of the staining matrix where no lyse was indicated. Samples were analyzed via a single platform using Flow-Count Fluorospheres on a Navios^™^ 10-color flow cytometer (Beckman Coulter). 7-Amino-Actinomycin-D (7-AAD) was used to exclude dead cells from analysis. At least 30,000–50,000 leucocytes (CD45^+^) were acquired and analyzed using the Navios Software. A standardized protocol (including cytometer's settings and gating strategy) was used to determine the leucocyte cell count, and the viability and frequency of the leucocyte subpopulations (CD4^+^ and CD8^+^ T cells, B cells, and NK cells). The protocol is being verified on a regular basis via attendance in proficiency testing and measurements of control cells (i.e., CD-Chex Plus™ BC; Streck, Omaha, NE). Fluorescence minus one (FMO) control was used to set the gates where no reference material was available (i.e., CD45RA, CD45RO, and CD62L). Plausibility checks were performed on fresh unmanipulated apheresis material. The settings of the apheresis sample and of the negative fraction sample after CD62L selection (mostly CD62L^−^) were used as the reference to set the gates for the target fraction after CD62L selection. Besides differentiation between CD4^+^CD3^+^ T helper and CD8^+^CD3^+^ cytotoxic T cells, the overall T cells were subdivided into naïve (T_N_ CD45RA^+^CD62L^+^), central memory (T_CM_ CD45RA^−^CD62L^+^), effector memory (T_EM_ CD45RA^−^CD62L^−^), CD45RA positive effector memory cells (T_EMRA_ CD45RA^+^CD62L^−^), and stem memory T cells (T_SCM_ CD45RA^+^CD45RO^+^CD62L^+^) as described in detail in the literature.^14,15^ The flow cytometer's fluidic stability and optical alignment were verified daily using Flow-Check^™^ Fluorospheres (Beckman Coulter).

In addition, the MACS Quant^®^ Analyzer 10 (Miltenyi Biotec) was used for both cell characterization and quantification of transduction efficacy. Cells were harvested and washed once in cold CliniMACS PBS/EDTA buffer supplemented with 0.5% human heat-inactivated AB serum (GemCell, West Sacramento, CA). After washing, cells were resuspended in staining mix containing the following antibody-fluorochrome conjugates (all Miltenyi Biotec) for the detection of the T cell phenotype after recovery: CD45-VioBlue, CD3-APC-Vio770, CD62L-APC, and CD45RO-FITC. For the detection of GFP expression, CD45-VioGreen and CD3-VioBlue were used 7 days after transduction. After 10 min of incubation, cells were washed, and flow cytometric acquisition was performed with the MACSQuant^®^ Analyzer 10 and analyzed with the MACSQuantify^™^ 2.5 software (Miltenyi Biotec). Propidium iodide was added to exclude dead cells. In addition, fluorescence microscopy was performed with a Zeiss Axiovert 200 M (Cellomics ArrayScan, HCS System; Zeiss, Oberkochen, Germany).

## Results

### Downregulation of CD62L on T cells due to freezing and thawing

**Figure 2.**
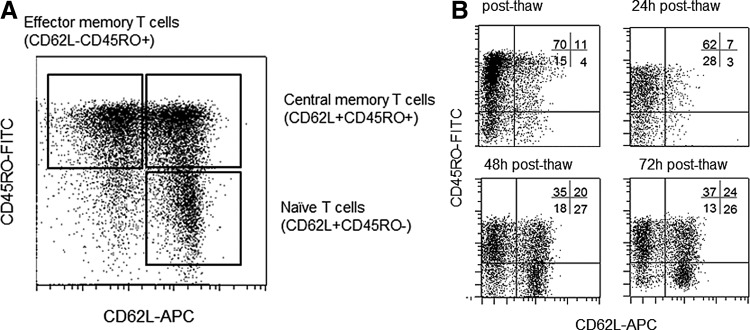
CD62L expression on naïve and central memory T cells is downregulated due to freezing and thawing. (**A)** Characterization of T cell phenotype in apheresis product of healthy donor using CD62L and CD45RO expression among all viable CD45^+^CD3^+^ cells. Naïve T cells were defined as CD3^+^CD62L^+^CD45RO^−^, central memory T cells as CD3^+^CD62L^+^CD45RO^−^, and effector memory T cells as CD3^+^CD62L^−^CD45RO^−^. (**B**) Apheresis product of a healthy donor was stored at –70°C, and thawed and recovered in TexMACS^™^ medium without supplements (37°C, 5% CO_2_). T cells were analyzed directly as well as 24, 48, and 72 h after thawing. Characterization of T cell phenotype was done via CD62L and CD45RO re-expression among all viable CD45^+^CD3^+^ cells.

In order to evaluate whether part of the apheresis or CD62L^+^ selected T cells can be cryopreserved for later cell processing including TCT, CD62L antigen expression on T cells was analyzed. The fresh apheresis product contains CD45RO^−^CD62L^+^ naïve T cells, CD45RO^+^CD62L^+^ central memory T cells, as well as CD45RO^+^CD62L^−^ effector memory and CD45RO^−^CD62L^−^ effector T cells (Fig. 2A). Freezing and thawing of the apheresis resulted in a clear downregulation of CD62L expression, which was restored during cultivation (gated on CD45^+^CD3^+^). The percentage of CD62L^+^ cells increased from 15% immediately after thawing to 50% after 72 h of cultivation (Fig. 2B). These experiments led to the decision to purify naïve and central memory T cells from fresh apheresis products and consequent cryopreservation.

### Study design

To evaluate the manufacturing process, three mock runs and one GFP run (donor 3_GFP_) for proof of principle were performed. Mock runs were carried out with fresh CD62L selected cells (donor 1, donor 2_fresh_) and cryopreserved CD62L selected cells (donor 2_cryo_). Apheresis of healthy donors was used as the starting material, and the cultivation time was 10–13 days.

### Automated clinical scale purification and expansion of CD62L^+^CD3^+^ T cells and in-process control

**Figure 3.**
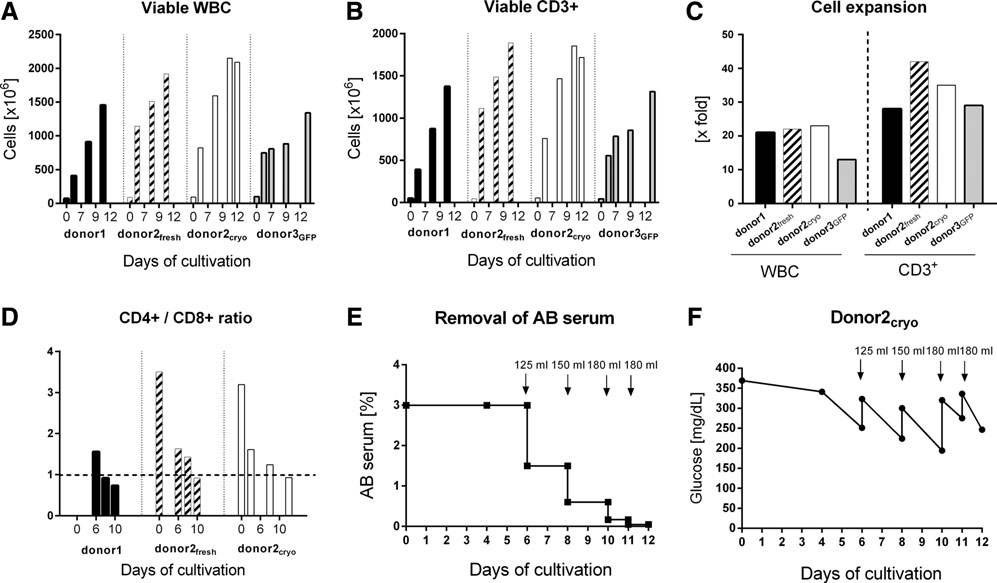
Clinical scale cell expansion of CD62L selected T cells in the Prodigy system (three mock runs and one green fluorescent protein [GFP] vector run). (**A**) Increase in absolute viable leucocytes on different days up to harvesting; CD45^+^ 7-AAD neg. cells are quantified with single platform flow cytometry analysis. WBC, white blood cells. (**B**) Absolute cell numbers of viable CD3^+^ lymphocytes on different days in culture. (**C**) Expansion rate from beginning of cultivation (day 0) up to harvesting (days 10–13) for both, viable WBC and viable CD3^+^ T cells. (**D**) Ratio of CD3^+^CD4^+^ and CD3^+^CD8^+^ lymphocytes at different days of culture of all mock runs (donor1, donor2_fresh_, and donor2_cryo_). (**E**) Removal of AB serum during T cell expansion caused by medium exchange as indicated (125 mL, 150 mL, and 180 mL = exchange of 125 mL, 150 mL, and 180 mL), shown for the mock run starting with cryopreserved CD62L-selected T cells (donor2_cryo_). (**F**) In-process control of glucose level during T cell expansion shown for the mock run (donor2_cryo_).

CD62L selection of three unstimulated leukapheresis products led to an overall recovery of CD3^+^ cells between 37.3 and 79.9 (median 66.8%). The recovery was calculated as the ratio between CD3^+^ cells after CD62L selection and CD3^+^ CD62L^+^ before selection due to the masking of the CD62L antigen by the selection reagent. While more than four out of five of selected cells were cryopreserved, only 70–100 × 10^6^ (median 91 × 10^6^) viable leucocytes containing 44–54 × 10^6^ (median 47 × 10^6^) viable CD3^+^ cells were further processed in the Prodigy system, leading to a final product containing 1,340–2,091 × 10^6^ (median 1,685 × 10^6^) viable leucocytes (viability >85%) and 1,313–1,889 × 10^6^ (median 1,547 × 10^6^) viable CD3^+^ cells after a cultivation time of 10–13 days (Fig. 3A and B). Accordingly, the expansion rate was 13- to 23-fold (median 22-fold) for viable leucocytes, and 28- to 42-fold (median 32-fold) for CD3^+^ T cells (Fig. 3C). Notably, this low variance includes the expansion of both fresh and cryopreserved CD62L selected cells (Fig. 3A–D).

During cultivation, the ratio of CD4^+^ and CD8^+^ cells among CD3^+^ T cells showed concordant patterns that followed similar kinetics, as shown in Fig. 3D for donor1, donor2_fresh_, and donor2_cryo_. The ratio between CD4^+^ and CD8^+^ cells decreased from 1.6 to 0.7 (donor1), from 3.5 to 0.9 (donor2_fresh_), and from 3.2 to 0.9 (donor2_cryo_), respectively.

Reduction of human AB serum during cultivation by media exchange from day 5 is shown in Fig. 3E. Starting with 3% (v/v) human AB serum, the final product contains 0.047% (v/v) only. For monitoring cell culture conditions, the glucose concentration was monitored as an in-process control, as shown in Fig. 3F. Concerning the supply with glucose, the schedule of feeding/medium exchange was sufficient not to fall below a critical level of 100 mg/dL.

### Cell composition during the manufacturing process

**Figure 4.**
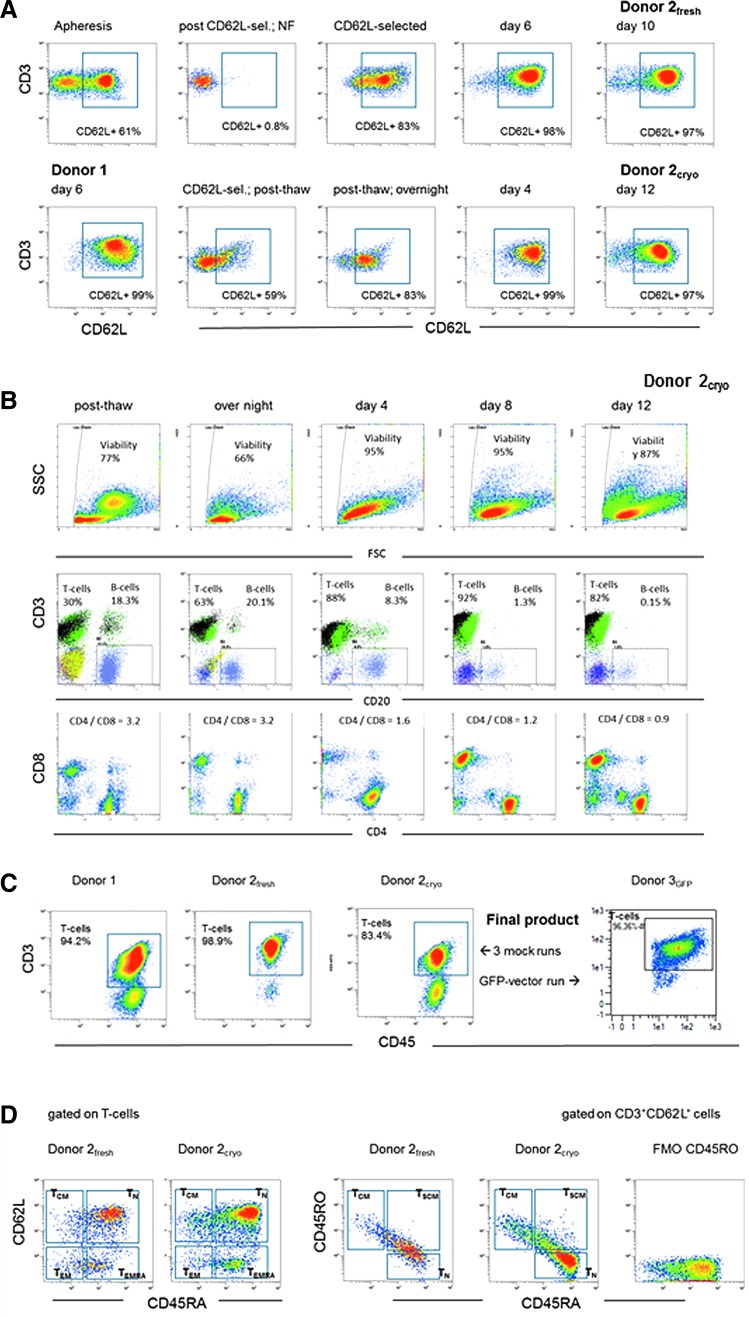
Flow cytometric analyses of CD62L purified T cells on different days during manufacturing in the Prodigy system. (**A**) Frequency of CD62L^+^ cells gated on viable T lymphocytes (7-AAD^−^CD45^+^CD3^+^). *Upper row*: starting material (apheresis of donor2), after CD62L-selection (non-target and target fraction), on days 6 and 10 (harvesting) of cultivation. *Lower row*: first plot—donor1 cells on day 6, plots 2–5—donor2_cryo_ on the day of thawing after 24 h recovery on days 4 and 12 (harvesting) of cultivation. NF, non-target fraction. (**B**) Cellular subpopulation during cultivation (donor2_cryo_). *Upper row*: forward (FSC) and side light-scatter (SSC) features of the cell suspension, plots gated on viable leucocytes (7-AAD^−^CD45^+^). *Middle row*: frequency of T cells (7-AAD^−^CD45^+^CD3^+^) and B cells (7-AAD^−^CD45^+^CD20^+^); plots gated on viable leucocytes (7-AAD^−^CD45^+^). *Lower row*: distribution (ratio) of CD4^+^ and CD8^+^ cells; plots gated on T lymphocytes. (**C**) Frequency of T cells (7-AAD^−^CD45^+^CD3^+^) in all four Prodigy products (at harvesting); plots gated on viable leucocytes (7-AAD^−^CD45^+^), including three mock runs and one run using GFP transduced T cells. (**D**) Phenotyping profile of the final product of donor2_fresh_ and donor2_cryo_ (flow cytometric analysis performed on cryopreserved/thawed product; cells analyzed 48 h after thawing). Distribution of naïve (T_N_ CD45RA^+^CD62L^+^), central memory (T_CM_ CD45RA^−^CD62L^+^), effector memory (T_EM_ CD45RA^−^CD62L^−^), CD45RA^+^ effector memory cells (T_EMRA_ CD45RA^+^CD62L^−^), and stem memory T cells (T_SCM_ CD45RA^+^CD45RO^+^CD62L^+^). Left panel gated on CD3^+^ cells; right panel gated on CD3^+^CD62L^+^ cells.

While approximately two thirds of the unmanipulated apheresis consisted of CD62L^+^ cells, the target fraction after CD62L enrichment contained >80% CD62L^+^ cells. The percentage of CD62L^+^ rose to >95% of the final product at day 10–13, as shown in Fig. 4A for donor2_fresh_ and donor2_cryo_. In this respect, it has to be noted that due to the masking of the CD62L receptor during immunomagnetic labeling, the quantification of enriched CD62L^+^CD3^+^ cells is impaired (Fig. 4A). Additionally, the downregulation of CD62L expression after cryopreservation/thawing adversely affects the precision of flow cytometric analysis of CD62L^+^ cells after selection/thawing (donor2_cryo_) in the first days of cultivation. Both effects disappeared during early cultivation, and the frequency of CD62L^+^ rose to 99% for donor1 (day 6), 98% for donor2_fresh_ (day 6), and 99% for donor2_cryo_ (day 4).

Overall, T cell purity of the final product of all four clinical-scale runs (three mock runs, one GFP run) was 83.4–98.9% CD3^+^CD45^+^ T cells (Fig. 4C). The predominant subpopulation displays a CD45RA^+^CD62L^+^ naïve (TN) phenotype (>75%) and small proportions of central memory CD45RA^−^CD62L^+^ (T_CM_), effector memory CD45RA^−^CD62L^−^ (T_EM_), and CD45RA positive effector memory cells (T_EMRA_), as exemplarily shown for donor2_fresh_ and donor2_cryo_ (Fig. 4D). The overall CD62L^+^CD45RA^+^ T cells can be further subdivided in true naïve CD45RA^+^CD45RO^−^CD62L^+^ and stem memory CD45RA^+^CD45RO^+^CD62L^+^ (T_SCM_) T cells (Fig. 4D).

Further details of cellular composition and characteristics during cultivation and at harvest are shown exemplarily for donor2_cryo_ (Fig. 4B). Immediately after thawing, the viability of leucocytes was 77%, which declined to 66% after overnight storage in cultivation bags (MACS GMP Cell Differentiation Bag-250, Miltenyi Biotec) incubated at 37°C. During further cultivation in the Prodigy system, viability increased to 95% on day 4 and day 8, and did not fall below 87% at harvesting on day 12 (Fig. 4B, first row). CD3^+^ T cell purity was 92% on day 8, and 82% at harvesting (Fig. 4B, middle row). The CD4/CD8 ratio among the T cells reversed from 3.2 immediately after thawing to 0.9 on day 12 (Fig. 4B, lower row). The other remaining cells were B cells and NK cells, respectively. During cultivation, the amount of CD20^+^ B cells declined continually from 20% to about 0.15% on day 12 (Fig. 4B, middle row).

### Stimulation and transduction of CD62L^+^ cells in the Prodigy system

**Figure 5.**
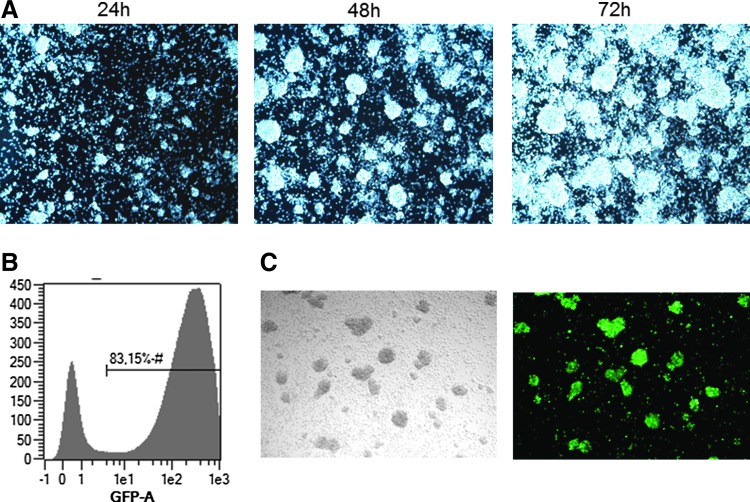
CD62L enriched T cells during expansion and transduction on day 1 with lentiviral vector encoding GFP. (**A**) CD62L-enriched T cells cultured in TexMACS^™^ GMP medium supplemented with IL-7 and IL-15 were activated using MACS GMP TransAct CD3/CD28 Kit (1:200 and 1:400). T cell clustering after TransAct stimulation. Pictures taken with the integrated microscope camera in the Prodigy system 24–72 h after start of stimulation. (**B**) Flow cytometric analysis of transduction efficacy on day 7 (MACS Quant Analyzer 10). (**C**) Fluorescence microscopy (Zeiss Axiovert 200 M, Cellomics ArrayScan, HCS System). Sample was taken from the Prodigy system and transferred to a 6-well culture plate. After resting the cells for 2 h at 37°C, 5% CO_2_ pictures were taken.

The stimulation of T cells via CD3 (first signal) and CD28 (second signal) leads to the clustering of the cells, which could be monitored by taking pictures with the Prodigy's integrated camera (Fig. 5A) during static culture until day 3. After cultivation was switched to dynamic mode by start of agitation of the chamber, clusters were dispersed. Transduction performed over a period of 48 h (from day 1 to day 3) with lentiviral vector containing GFP resulted in a transduction efficiency of 83%, as shown by flow cytometric analysis and fluorescence microscopy (Fig 5B and C).

## Discussion

The growing interest in the field of gene-modified T cells, especially the promising results with CAR T cells in several clinical studies, requires continuous improvement in manufacturing protocols toward automatization. In this respect, the aim of this study was to establish and demonstrate the proof of principle of a closed, GMP-compliant, clinical-scale protocol for the complex manufacturing of purified, transduced, and expanded naïve and central memory T cells over a period of 2 weeks. Manufacturing of genetically engineered T cells combines different steps of separation, stimulation, transduction, and long-term cultivation. To date, those parts are often performed in consecutive hands-on, offline steps in not consistently closed systems. The recently introduced Prodigy device to be used with closed sterile tubing sets fitted with a centrifuge and cultivation unit allows for the aseptic GMP-compliant integration of liquid management, immunomagnetic separation, cultivation, sampling, and formulation, including in-process microphotography of cellular therapeutics and intermediates in one integrated system.^16^

So far, Stroncek *et al*.^11^ and Spohn *et al*.^17^ have shown the successful use of this device for immunomagnetic CD34 selection with similar results using the automated system compared to the standard semi-automated CliniMACS^®^ Plus system in three runs. This is supported by Huemmer *et al*. reporting equivalent results for one split run.^10^ Bunos *et al*.^12^ demonstrated the enrichment of CMV-reactive T cells from donor apheresis products in five clinical scale runs reaching 64–93% purity of CD3^+^ T cells. In addition, the Prodigy system was successfully used for a 2-week cell cultivation with a >850-fold expansion of NK cells using IL-2 and irradiated clinical-grade Epstein–Barr virus–transformed lymphoblastoid feeder cells, as shown by Granzin *et al*.^13^

Based on the knowledge that effector memory T cells undergo apoptosis following adoptive transfer and do not persist, whereas central memory and especially naïve T cells persist for years,^18^ a CD62L selection was chosen to purify both central memory and naïve T cells. Purification of memory T cells for adaptive transfer using anti-CD62L magnetic beads has been successfully shown by Casati *et al*.^19^

In addition, this study demonstrated that cryopreservation and thawing of apheresis products leads to a downregulation of CD62L expression on CD3^+^ T cells. It was concluded that fresh apheresis products should be purified immunomagnetically with CD62L beads freshly followed by cryopreservation of the target fraction, rather than using a cryopreserved thawed apheresis product as the starting material. Most importantly, the present data clearly demonstrate very similar expansion of the overall CD3^+^ T cells comparing freshly CD62L-selected naïve and effector memory T cells to cryopreserved thawed ones. All clinical scale runs resulted in a nearly identical conversion of the CD4/CD8 ratio. This is in line with the results of Hollatz^20^ using bead-coupled or soluble antibodies + IL-2. In addition, the viability and expansion rate of the overall CD3^+^ T cells at harvest was equal between cryopreserved and fresh CD62L-selected T cells. This clearly points to purified T cells being easily stored as an intermediate for later use in clinical studies. The apparent low purity of CD62L cells directly after enrichment is most likely due to the use of the same mAb clones for magnetic labeling and detection. CD62L purity recovered from 83% post enrichment to >97% as early as day 4 when cells started to divide. Interestingly, the final product contained mainly naïve CD45RA^+^CD45RO^−^CD62L^+^ and the stem memory CD45RA^+^CD45RO^+^CD62L^+^ (T_SCM_) T cells, which are important for long-living active T cells. These results are in accordance with the observation by Cieri *et al*.^15^ The reason for the formation of naïve and stem memory T cells is thought to be due to the CD3/CD28 engagement in combination with IL-7/IL-15 culturing used in both studies, which differ to the cytokine combination of other trials in the literature.

In addition to the four mock/GFP runs, a fifth run was also performed in the Prodigy system using a well-characterized CD19 CAR vector (Milone^21^). This run resulted in functional CAR T cells displaying killing activity against CD19^+^ JeKo-1 cells (ATCC CRL 3006; data not shown), which has to be confirmed and verified in further experiments. Nevertheless, the observation has been confirmed very recently in a publication by Mock *et al*.^22^ They successfully demonstrated the Prodigy system's eligibility for the manufacture of functional CD19 CAR T cells, leaving out initial immunoselection under an otherwise comparable process regimen. Inoculation of 7.5 × 10^7^–1.0 × 10^8^ lymphocytes resulted in 16.2 (±7.9)-fold T cell expansion until day 8–10, corresponding to a yield of 15.8 ± 6.4 × 10^8^ CD3^+^ cells, 47.6 ± 14.9% of which were CD19-CAR^+^. The mean purity of T cells in the final product was 92.3%. Irrespective of initial immunoselection, these results appear to be qualitatively and quantitatively similar to those in the present study. Given the Prodigy system's limited capacity regarding cultivation volume, it may be reasonably assumed that a further refinement of the initial immunoselection, including the questioning of CD62L as the target antigen of choice, will potentially improve process yield.

The clinical manufacture of gene-modified cells is complex, and consists of several process steps requiring relevant hands-on time by experienced operators. To reduce error and increase process precision and robustness, separate semi/automatic devices were developed for the enrichment/selection (CliniMACS^®^ Plus, Miltenyi Biotec; Dynal ClinExVivo magnetic particle concentrator, Thermo Fisher Scientific, Inc., Waltham, MA), washing (Cobe cell processor; Terumo BCT, Inc., Lakewood, CO), and expansion (Xuri Cell expansion system/Wave bioreactor, G-Rex technology; Wilson Wolf Manufacturing Co., New Brighton, MN) of cells. The implementation of semi-closed culture systems including GMP-compatible culture bags, tubings, connectors, and other accessories, and the availability of separation and activation reagents, media, cytokines, and buffers meeting the requirements of USP<1043> on ancillary materials enables the cultivation, activation, and expansion of cells as ATMPs. Sequential offline processing using isolated semi/automated platforms proved to be suitable for the manufacture of gene-modified CAR T cells or of T cell subtypes for adoptive cell therapy of cancer patients as investigational medicinal products for successfully evaluation in Phase I/II trials.^18,19,23–25^ This is currently reviewed by Wang *et al*.^26^ and Kim *et al*.^27^

To move forward in the direction of process integration, the performance of the Prodigy system was evaluated for the manufacture of those CD62L-selected T cells to be used for future gene therapy trials. The multi-step manufacturing process comprising of CD62L selection, stimulation, transduction, expansion, and formulation of the final product was performed automatically with one device. Consistent expansion rates were achieved with fresh as well as cryopreserved CD62L^+^ T cells. Regulatory aspects include the robustness of the manufacturing process, the consistency in composition of the transduced and expanded T cell products, and the absence of microbial contamination. Although hands-on operation was required during the clinical-scale runs such as dis/connecting media reservoirs and waste and sample pouches, the microbiological controls of intermediates and product by aerobic and anaerobic blood culture bottles remained inconspicuous. Glucose, used as a surrogate for nutrient limitation affecting cell growth and reactor productivity,^28^ always stayed above the critical level of 100 mg/dL in all runs.

In summary, this study demonstrated that a complex process including cell purification, activation, transduction, washing, cell feeding, and cell expansion can be integrated in one GMP-compliant, automated, and closed system allowing for robust cell manipulation leading to uniform gene-modified cellular products and allowing for harmonized manufacturing protocols for CAR T cells, for example. However, the refinement of the immunoselection step, as well as the proof of activity of retargeted cells *in vitro* and *in vivo*, will be the subject of further research.
